# Seven-year change of prevalence, clinical risk factors, and mortality of patients with carbapenem-resistant *Klebsiella pneumoniae* bloodstream infection in a Chinese teaching hospital: a case-case-control study

**DOI:** 10.3389/fmicb.2025.1531984

**Published:** 2025-03-19

**Authors:** Haifang Kong, Yong Liu, Ling Yang, Qianqian Chen, Yanchun Li, Zhidong Hu, Xuequan Feng, Yamin Chai, Zuoliang Dong

**Affiliations:** ^1^Department of Laboratory Medicine, Tianjin Medical University General Hospital, Tianjin, China; ^2^Tianjin First Central Hospital of Nankai University, Tianjin, China

**Keywords:** risk factors, carbapenem resistance, *Klebsiella pneumoniae*, bloodstream infection, case-case-control study

## Abstract

Carbapenem-resistant *Klebsiella pneumoniae* bloodstream infection (CRKP-BSI) is a major public health threat worldwide. CRKP-BSI is associated with poor outcomes, elevated morbidity and mortality, and high healthcare costs. Therefore, the identification of risk factors for CRKP-BSI and mortality are critical for preventing and controlling CRKP in hospitals. This retrospective case-case-control study was conducted at General Hospital of Tianjin Medical University, a tertiary teaching hospital, from 2017 to 2023. It included 105 patients with CRKP-BSI (case group 1) and matched 105 patients with carbapenem-susceptible *K. pneumoniae* bloodstream infection (CSKP-BSI) (case group 2). The control group was selected at a ratio of 1:1:1 (case group 1: case group 2: control) from patients with a positive blood culture (except for *K. pneumoniae* infection) to analyze risk factors associated with the two case groups and compare the 30-day survival curves using multivariable logistic regression and Kaplan-Meier analyses. Multivariate analysis revealed that liver disease was a risk factor for *K. pneumoniae*-BSI, and exposure to carbapenem [odds ratio (OR) = 3.24], tigecycline (OR = 3.43), and glucocorticoids (OR = 4.64) were independent risk factors for CRKP-BSI. The 30-day mortality of the CRKP-BSI group was 30.5%, and patient groups, respiratory diseases (HR = 3.52), use of 3rd-generation cephalosporins (HR = 1.92), mechanical ventilation (HR = 2.14), and central venous catheter insertion (HR = 2.85) were independent risk factors, whereas a shorter length of hospitalization was a protective factor for 30-day mortality. The in-hospital mortality in the CRKP-BSI group was 55.2%, and arterial catheter use (OR = 3.76) was an independent risk factor for in-hospital mortality. Several factors were identified to contribute to the development of CRKP-BSI. CRKP isolates were resistant to most antibiotics. Reducing CRKP-BSI-related mortality requires comprehensive consideration of underlying diseases, judicious antibiotic use, and invasive procedures. The high morbidity, mortality, along with the limited therapeutic options for CRKP-BSI, underscore the need for improved detection, identification of risk factors to develop effective preventive measures, and development of novel agents with reliable clinical efficacy against CRKP.

## 1 Introduction

Carbapenems, a class of broad-spectrum beta-lactam antibiotics, are the primary treatment choice for severe infections caused by multidrug-resistant *Enterobacteriaceae*. Carbapenem-resistant *Klebsiella pneumoniae* (CRKP) was first identified in northeastern USA in the early 2000s (Tzouvelekis et al., 2012); however, with the increasing use of carbapenems in hospitals, the prevalence of CRKP is increasing worldwide and has constituted a serious threat to public health (Bonomo et al., 2018; David et al., 2019; Reyes et al., 2019). In Europe, the resistance rate to CRKP has been reported to be as high as 39.7% (David et al., 2019). In Eastern Mediterranean region, *Klebsiella pneumoniae* (*K. pneumoniae*) accounts for over 50% of infections (Prestinaci et al., 2015). According to data from the China Antimicrobial Surveillance Network (CHINET), the resistance rates of *K. pneumoniae* to imipenem and meropenem have increased steadily from 3 and 2.9% in 2015 to 24.4 and 25% in 2023, respectively (CHINET, 2023). In 2008, the World Health Organization (WHO) classified CRKP as a critical priority on the global priority list of antimicrobial-resistant bacteria (Tacconelli et al., 2018). In 2009, the Centers for Disease Control and Prevention (CDC) classified 18 common multidrug-resistant bacteria into three categories, designating CRKP as an urgent threat to human health (CDC., 2019). The mortality of patients with CRKP bloodstream infection (CRKP-BSI) is significantly high (Xu et al., 2017; Zhu et al., 2020), and BSI has become a major global medical burden (Markwart et al., 2020). The incidence of CRKP-BSI can increase mortality and prolong the length of hospitalization (Schwaber et al., 2008; Stewardson et al., 2019), which is an important clinical threat.

Currently, carbapenemase production is the primary mechanism of resistance in CRKP, with carbapenemases classified into class A (represented by KPC), class B (e.g., NDM, VIM, and IMP), and class D (primarily OXA-48). Overexpression of efflux pumps and loss of outer membrane porins also contribute to the development of resistance. *Klebsiella pneumoniae* carbapenemase (KPC) has emerged as the most prevalent carbapenemase in the United States and Europe (Grundmann et al., 2017; Temkin et al., 2014), and KPC-2 (64.6%) has become the most common carbapenemase in CRKP in China (Han et al., 2020). Plasmid-encoded carbapenemases often carry additional genes that confer resistance to other antibiotics, which can easily spread among bacteria, resulting in outbreaks and limited treatment options. Only a few antibiotics, such as tigecycline and polymyxins, remain effective against CRKP. Although novel antibiotics, such as ceftazidime-avibactam, have emerged for the treatment of CRE infections, they remain ineffective against New Delhi metallo-beta-lactamase (NDM)-producing CRKP (Abe et al., 2022; Chen Y. et al., 2022). Therefore, understanding the risk factors associated with the causes and outcomes of CRKP-BSI is required to reduce its incidence and initiate appropriate therapies.

Several studies have investigated the risk factors for CRKP infection. However, some of these studies employed a case-control design, comparing a CRKP group with a carbapenem-susceptible *Klebsiella pneumoniae* (CSKP) group. Such a case-control design has an intrinsic flaw as it is based on the “replacement scenario” assumption, in which each patient is assumed to be infected either by antimicrobial-resistant organisms or antimicrobial-susceptible counterparts; however, the patient may be infected by other pathogens or not infected at all. CSKP infections account for only a small proportion of hospitalized cases, making them unrepresentative (Evans and Harris, 2017; Harris et al., 2001; Kaier and Frank, 2013). Therefore, an improved design is required to further elucidate the risk factors for CRKP infection. In this study, we employed a case-case-control design to evaluate the risk factors for CRKP-BSI, providing a foundation for effective control strategies.

## 2 Materials and methods

### 2.1 Study design and Patient population

**FIGURE 1:**
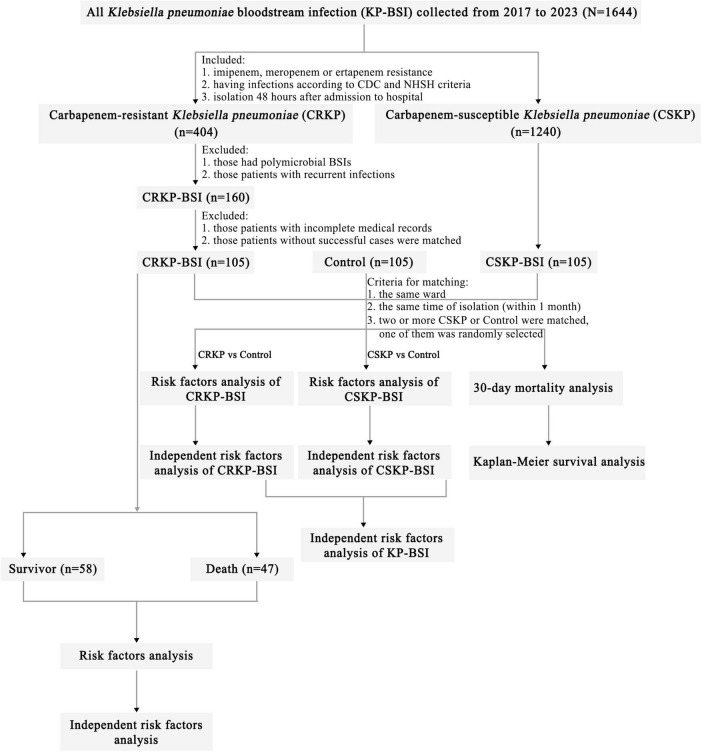
Flowchart of sample selection, comparison, and value. From a total of 1,644 samples, 105 CRKP isolates were selected for further analysis. A matched CSKP group and control group were used to analyze the risk factors for CRKP-BSI and CSKP-BSI, from which the risk factors for KP-BSI were obtained. Risk factors for 30-day mortality in CRKP-BSI were also analyzed. Based on the outcome of hospitalized patients, the 105 CRKP isolates were divided into survivors and death to analyze the risk factors for in-hospital mortality. Abbreviations: CRKP-BSI, carbapenem-resistant *Klebsiella pneumoniae* bloodstream infection; CSKP, carbapenem-susceptible *Klebsiella pneumoniae* bloodstream infection; CDC, Centers for Disease Control and Prevention; NHSN, National Healthcare Safety Network.

We performed a single-center, 7-year, continuous study of CRKP-BSI strains using specific inclusion and exclusion criteria (Figure 1). A total of 105 clinical CRKP-BSI isolates were collected from Tianjin Medical University General Hospital, a 2,468-bed tertiary-care hospital in China, from January 2017 to December 2023. According to the CDC, BSIs are defined as the isolation of pathogenic organisms from blood culture tests (Calandra and Cohen, 2005). Only the first CRKP or CSKP isolate from the bloodstream of each patient was included. Nosocomial CRKP, CSKP, or control group infections were defined as isolates obtained ≥ 48 hours after hospital admission.

The case group included hospitalized patients infected with CRKP-BSI or CSKP-BSI. Inpatients who met any of the following criteria were excluded: (1) patients infected with CRKP-BSI and/or CSKP-BSI before or within 2 days of hospital admission, (2) patients with polymicrobial BSIs, (3) patients with recurrent infections; (4) patients with incomplete medical records, and (5) patients infected without CSKP-BSI at the identical ward and timeframe as the CRKP-BSI cases.

### 2.2 Case definitions

Based on previous studies (Kaye et al., 2005; Ng et al., 2014), we designed the case-case-control study. This study comprised two case-control comparisons, in which inpatients infected with CRKP-BSI and CSKP-BSI were compared to a matched inpatients control group, respectively. The CSKP and control groups were matched to the CRKP group in the same ward as the source population during the same period (within 1 month) as the CRKP group. Patients in the CSKP and control groups were randomly selected when two or more cases were matched.

The CRKP group comprised patients with positive blood cultures, and isolates resistant to any carbapenem (imipenem, meropenem, and ertapenem) were considered resistant.

The CSKP group comprised patients with positive blood cultures and was defined as having *K. pneumoniae* susceptible to meropenem, imipenem, and ertapenem.

The matched control group comprised patients with positive blood cultures who have been confirmed bacteremia (except for *K. pneumoniae* infection). The control group included patients infected with Gram-positive bacteria, Gram-negative bacteria (except *K. pneumoniae*), and fungal bloodstream infections.

### 2.3 Antimicrobial susceptibility testing

Blood samples were cultured using the Bactec™ FX 50 systems (Becton Dickinson, Microbiology Systems, Cockeysville, MD, United States). All isolates were identified using matrix-assisted laser desorption ionization time-of-flight mass spectrometry (BioMérieux, France). Vitek2-compact automated microbiology system (BioMérieux, France) was used for antimicrobial susceptibility testing. Carbapenem (imipenem and/or meropenem) resistance was confirmed using the Kirby–Bauer (K–B) method (CLSI, 2023).The Clinical and Laboratory Standards Institute (CLSI-M100) document was used to interpret the antimicrobial susceptibility testing (CLSI, 2023). *E. coli* ATCC25922 and *Pseudomonas aeruginosa* ATCC27853 were used as control strains for antimicrobial susceptibility testing.

### 2.4 Data collection

We reviewed the medical records and collected patient information according to previous studies (Chen J. et al., 2022; Kong et al., 2024; Ng et al., 2014; Palacios-Baena et al., 2021; Stuever et al., 2022; Zhang et al., 2021). The epidemiology and clinical data of patients were collected, including the department, sex, age, age > 65 years, underlying diseases (respiratory system, liver, urinary system, circulatory system, digestive system, and central nervous diseases; diabetes mellitus, malignancies); antibiotics (3rd-generation cephalosporins, β-lactam inhibitor compounds, carbapenem, aminoglycosides, quinolones, tigecycline, and macrolides), and glucocorticoids, within 3 months before CRKP-BSI. Surgical history and invasive procedures (mechanical ventilation, central venous catheter, arterial, and urinary catheters, drainage, and gastric tubes), within 1 month before CRKP-BSI; mortality, related to hospitalization (hospital stays prior CRKP isolation, ICU stays within 3 months before a positive blood culture, length of hospital stays). Acute Physiology and Chronic Health Evaluation II (APACHE II) and Sequential Organ Failure Assessment (SOFA) scores are used to calculate the severity of the disease within 24 h following the onset of BSI.

The clinical outcomes were defined as follows: 30-day mortality (within 30 days of the first positive blood culture), in-hospital mortality (death during hospitalization after the first positive blood culture), and length of hospital stays (duration from admission to hospital-to-hospital discharge).

### 2.5 Statistical analysis

Non-normally distributed continuous variables are presented as medians with interquartile ranges (IQRs) and were compared using the Mann–Whitney U-test. Categorical variables are presented as numbers and percentages, and were compared using the chi-square test or Fisher’s exact test. Analysis of variance (ANOVA) was used for comparison among the three groups. Univariate analyses were performed for each variable, and those with a *p* < 0.05 were included in a multivariate logistic regression analysis. Statistical significance was set at *p* < 0.05, and SPSS 26.0 and GraphPad Prism 10 were used for statistical analyses.

## 3 Results

### 3.1 Incidence and mortality of CRKP-BSI over the past 7 years

**FIGURE 2:**
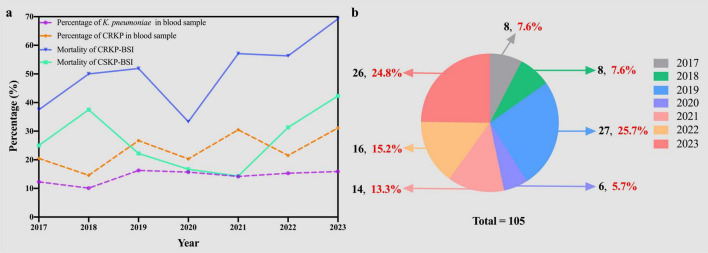
**(A)** Trends in prevalence and mortality of *Klebsiella pneumoniae* bloodstream infections. **(B)** The number (black) and proportion (red) of CRKP-BSI strains isolated over the seven-year study period. KP, *Klebsiella pneumoniae*; CRKP, carbapenem-resistant *Klebsiella pneumoniae*; CSKP, carbapenem-susceptible *Klebsiella pneumoniae*; BSI, bloodstream infection.

During the 7-year study period, only 105 patients met the inclusion criteria, resulting in 105 episodes of CRKP-BSI included in the analysis. Most patients infected with CRKP-BSI were admitted to the intensive care unit (ICU) (48.6%, 51/105), followed by stays in general medical (38.1%, 40/105) and surgical (13.3%, 14/105) wards. The percentage of *K. pneumoniae* in the blood samples from 2017 to 2023 fluctuated from 12.3 to 15.9% (12.3% in 2017, 10.1% in 2018, 16.3% in 2019, 15.7% in 2020, 14.2% in 2021, 15.3% in 2022, and 15.9% in 2023). The percentages of CRKP-BSI in *K. pneumoniae*-BSI from 2017 to 2023 were 20.5, 14.6, 26.7, 20.3, 30.5, 21.5, and 31.1%, respectively, thus increasing from 20.5 to 31.1% (Figure 2A). In addition, the in-hospital mortality of patients with CRKP-BSI was 55.2%, which was higher than that of patients with CSKP-BSI (55.2 vs. 28.6%, *p* < 0.001). The in-hospital mortality of CRKP-BSI and CSKP-BSI increased from 37.5 and 25%, respectively, in 2017 to 69.2 and 42.3% in 023 (Figure 2A). The number and proportion of isolated cases of CRKP-BSI annually are shown in Figure 2B, most of the strains were isolated in 2019 and 2023.

### 3.2 Antimicrobial susceptibility of CRKP-BSI and CSKP-BSI

The antibiotic susceptibility patterns of CRKP-BSI and CSKP-BSI isolates are shown in Table 1. All CRKP-BSI isolates were resistant to cefuroxime, and ceftriaxone. The resistance rate of CRKP-BSI isolates to amoxicillin/clavulanic acid, cefoperazone/sulbactam, piperacillin/tazobactam, ceftazidime, cefepime, aztreonam, gentamicin, levofloxacin, ciprofloxacin was higher than 80%, whereas the resistance rate to colistin and tigecycline was low.

**TABLE 1:** The antibiotic resistance of CRKP-BSI and CSKP-BSI groups.

Antibiotics	CRKP-BSI (*n* = 105)	CSKP-BSI (*n* = 105)	χ^2^	*p*
Amoxicillin/clavulanic acid	103/105 (98.1%)	13/105 (12.4%)	156.0	<0.001
Cefoperazone/sulbactam	102/105 (97.1%)	7/105 (6.7%)	172.2	<0.001
Piperacillin/tazobactam	104/105 (99.0%)	8/105 (7.6%)	176.3	<0.001
Cefuroxime	105/105 (100%)	32/105 (30.5%)	111.9	<0.001
Ceftazidime	103/105 (98.1%)	19/105 (18.1%)	138.0	<0.001
Ceftriaxone	105/105 (100%)	29/105 (27.6%)	119.1	<0.001
Cefepime	104/105 (99.0%)	19/105 (18.1%)	141.8	<0.001
Aztreonam	101/105 (96.2%)	19/105 (18.1%)	130.7	<0.001
Amikacin	70/105 (66.7%)	1/105 (1.0%)	101.3	<0.001
Gentamicin	92/105 (87.6%)	18/105 (17.1%)	104.5	0.001
Tobramycin	78/105 (74.3%)	7/105 (6.7%)	99.6	<0.001
Ciprofloxacin	96/105 (91.4%)	33/105 (31.4%)	79.8	<0.001
Levofloxacin	98/105 (93.3%)	19/105 (18.1%)	120.4	<0.001
Trimethoprim/sulfamethoxazole	60/105 (57.1%)	28/105 (26.7%)	20.0	<0.001
Colistin	1/105 (1.0)	0/105 (0.0)	1.0	0.3
Tigecycline	29/105 (27.6)	9/105 (8.6)	12.9	<0.001

CRKP-BSI, carbapenem-resistant *Klebsiella pneumoniae* bloodstream infection; CSKP-BSI, carbapenem-susceptible *Klebsiella pneumoniae* bloodstream infection.

### 3.3 The CRKP-BSI group compared to the control group

Descriptive statistics of the study groups are shown in Table 2. Differences among study groups were identified for prior hospital stays (χ^2^ = 5.35, *p* = 0.005), ICU stays (χ^2^ = 8.39, *p* < 0.001), and length of hospital stays (LOS) (χ^2^ = 4.65, *p* = 0.01).

**TABLE 2:** Demographic characteristics of 105 patients with CRKP-BSI, 105 patients with CSKP-BSI, and 105 control patients at a large tertiary-care hospital.

	CRKP-BSI [n (%)]	CSKP-BSI [n (%)]	Control [n (%)]	χ^2^/F	*P*
Sex, male	76 (72.4)	67 (63.8)	65 (61.9)	2.92	0.23
Age (years), median (IQR)	70 (58, 83)	68 (52, 80)	72 (61, 83)	1.28	0.28
Age > 65	65 (61.9)	58 (55.2)	69 (65.7)	2.48	0.29
APACHE II, median (IQR)	23 (21, 34)	18 (16, 36)	22.5 (18, 25)	0.86	0.44
SOFA, median (IQR)	10 (4.5, 11.5)	5 (5, 12.5)	10.5 (6.5, 11.5)	0.36	0.70
Prior hospital stays, median (IQR)	23 (12.5, 43)	12 (4, 25.5)	12 (4, 25.5)	5.35	0.005
ICU stays, median (IQR)	12 (0, 34.5)	0 (0, 11.0)	0 (0, 15)	8.39	<0.001
LOS (days), median (IQR)	41 (20, 66)	32 (18, 50.5)	34 (16, 70)	4.65	0.01

For non-normally distributed quantitative data, they were expressed as median (IQR); Qualitative data were expressed as number and percentage [n (%)]. CRKP-BSI, carbapenem-resistant *Klebsiella pneumoniae* bloodstream infection; CSKP-BSI, carbapenem-susceptible *Klebsiella pneumoniae* bloodstream infection; APACHE II: Acute Physiology and Chronic Health Evaluation II; SOFA, Sequential Organ Failure Assessment LOS; length of hospital stays; IQR, interquartile range.

The results of the univariate analyses comparing the clinical characteristics of patients infected with CRKP-BSIs and control patients are listed in Table 3. The variables associated with CRKP-BSI were as follows: respiratory diseases (OR = 2.51; 95%CI, 1.34–4.70; *p* = 0.004), liver diseases (OR = 1.78; 95%CI, 1.03–3.08; *p* = 0.04), and malignancies (OR = 2.07; 95%CI, 1.06–4.05; *p* = 0.03); exposure to antibiotics, including 3rd-generation cephalosporins (OR = 1.80; 95%CI, 1.02–3.20; *p* = 0.04), β-lactam inhibitor compounds (OR = 2.24; 95%CI, 1.27–3.94; *p* = 0.005),carbapenems (OR = 6.93; 95%CI, 3.78–12.73; *p* < 0.001), quinolones (OR = 2.48; 95%CI, 1.28–4.80; *p* = 0.006), tigecycline (OR = 4.00; 95%CI, 2.01–7.95; *p* < 0.001), glycopeptides (OR = 5.42; 95%CI, 1.16–25.38; *p* = 0.02), and glucocorticoids (OR = 3.92; 95%CI, 2.15–7.15; *p* < 0.001), within 3 months before a positive blood culture; surgical history (OR = 2.51; 95%CI, 1.43–4.42; *p* = 0.001) and invasive procedures, including mechanical ventilation (OR = 2.99; 95%CI, 1.71–5.25; *p* < 0.001), central venous catheter insertion (OR = 3.95; 95%CI, 2.19–7.13; *p* < 0.001), urinary catheter insertion (OR = 2.50; 95%CI, 1.38–4.51; *p* = 0.002), drainage tube insertion (OR = 2.44; 95%CI, 1.24–4.79; *p* = 0.009), and gastric tube insertion (OR = 2.10; 95%CI, 1.21–3.66; *p* = 0.008), within 1 month before a positive blood culture; prior hospital stays (*p* < 0.001), and ICU stays (*p* = 0.005). Multivariate conditional logistic regression analysis indicated that liver diseases (OR = 2.37; 95%CI, 1.21–5.00; *p* = 0.02) and exposure to carbapenems (OR = 3.24; 95%CI, 1.51–6.95; *p* = 0.003), tigecycline (OR = 3.43; 95%CI, 1.40–8.41; *p* = 0.007) and glucocorticoids (OR = 4.64; 95%CI, 2.15–10.02; *p* < 0.001), within 3 months before a positive blood culture, were independent risk factors for CRKP-BSI (Table 4).

**TABLE 3:** Univariate analyses of the risk factors for CRKP-BSI compared to the control group (*N* = 105).

Variable	CRKP-BSI [n (%)]	Control [n (%)]	χ^2^/U	OR	95%CI	*p*
**Demographic characteristics**						
Sex, male	76 (72.4)	65 (61.9)	2.61	1.17	(0.97, 1.42)	0.11
**Underlying disorder**						
Respiratory diseases	85 (81.0)	66 (62.9)	8.51	2.51	(1.34, 4.70)	0.004
Liver diseases	63 (60.0)	48 (45.7)	4.30	1.78	(1.03, 3.08)	0.04
Urinary system diseases	55 (52.4)	49 (46.7)	0.69	1.26	(0.73, 2.16)	0.41
Circulatory diseases	70 (66.7)	78 (74.3)	1.47	0.69	(0.38, 1.26)	0.23
Central nervous diseases	38 (36.2)	37 (35.2)	0.21	1.04	(0.59, 1.83)	0.89
Digestive system diseases	31 (29.5)	22 (21.0)	2.04	1.58	(0.84, 2.97)	0.15
Diabetes mellitus	30 (28.6)	37 (35.2)	1.07	0.74	(0.41, 1.32)	0.30
Malignancies	30 (28.6)	17 (16.2)	4.63	2.07	(1.06, 4.05)	0.03
**Antibiotics exposure within 3 months before CRKP-BSI**						
3rd-generation cephalosporins	44 (41.9)	30 (28.6)	4.09	1.80	(1.02, 3.20)	0.04
β-lactam inhibitor	73 (69.5)	53 (50.5)	7.94	2.24	(1.27, 3.94)	0.005
Carbapenem	73 (69.5)	26 (24.8)	42.21	6.93	(3.78, 12.73)	<0.001
Aminoglycosides	1 (1.0)	3 (2.9)	1.02	0.33	(0.03, 3.20)	0.31
Quinolones	34 (32.4)	17 (16.2)	7.48	2.48	(1.28, 4.80)	0.006
Tigecycline	40 (38.1)	14 (13.3)	16.85	4.00	(2.01, 7.95)	<0.001
Glycopeptides	10 (9.5)	2 (1.9)	5.66	5.42	(1.16, 25.38)	0.02
Antifungal agents	22 (21.0)	13 (12.4)	2.78	1.88	(0.89, 3.96)	0.10
Glucocorticoid	55 (52.4)	23 (21.9)	20.89	3.92	(2.15, 7.15)	<0.001
**Surgical history and invasive procedures within 1 month before CRKP-BSI**						
Surgical history	55 (52.4)	32 (30.5)	10.38	2.51	(1.43, 4.42)	0.001
Mechanical ventilation	64 (61.0)	36 (34.3)	14.97	2.99	(1.71, 5.25)	<0.001
Central venous catheter insertion	80 (76.9)	47 (44.8)	21.70	3.95	(2.19, 7.13)	<0.001
Arterial catheters	52 (49.5)	44 (41.9)	1.23	1.36	(0.79, 2.35)	0.27
Urinary catheter insertion	80 (76.2)	59 (56.2)	9.38	2.50	(1.38, 4.51)	0.002
Drainage tube insertion	32 (30.5)	16 (15.2)	6.91	2.44	(1.24, 4.79)	0.009
Gastric tube insertion	68 (64.8)	49 (46.7)	6.97	2.10	(1.21, 3.66)	0.008
**Related to hospitalization**						
Prior hospital stays median (IQR)	23 (12.5, 43)	12 (4, 25.5)	3605	—	—	<0.001
ICU stay, median (IQR)	12 (0, 34.5)	0 (0, 15)	4317	—	—	0.005
LOS (days), median (IQR)	41 (20, 66)	34 (16, 70)	4916	—	—	0.18
Death	58 (55.2)	26 (24.8)	20.32	3.75	(2.09, 6.74)	<0.001

For non-normally distributed quantitative data, they were expressed as median (IQR); Qualitative data were expressed as number and percentage [n (%)]. β-lactam inhibitor referred to β-lactamase inhibitors (piperacillin tazobactam and cefoperazone sulbactam), and carbapenem referred to one of the carbapenems (imipenem, meropenem, doripenem). CRKP-BSI, carbapenem-resistant *Klebsiella pneumoniae* bloodstream infection; ICU, intensive care unit; LOS, length of hospital stays; IQR, interquartile range; —, no result.

**TABLE 4:** Multivariate analyses of the independent risk factors for CRKP-BSI and CSKP-BSI compared to the control group.

Variable	β	SE	Wald (χ^2^)	OR	*95%CI*	*p*
**CRKP-BSI vs. control group**						
Liver diseases	0.86	0.38	5.12	2.37	1.12∼5.00	0.02
Carbapenem	1.17	0.39	9.07	3.24	1.51∼6.95	0.003
Tigecycline	1.23	0.46	7.23	3.43	1.40∼8.41	0.007
Glucocorticoids	1.53	0.39	15.21	4.64	2.15∼10.02	< 0.001
**CSKP-BSI vs. control group**						
Liver diseases	0.60	0.29	4.22	1.82	1.03∼3.22	0.04
Malignancy	0.87	0.35	6.10	2.38	1.20∼4.75	0.01
**Death vs. survivors in the CRKP-BSI group**						
Arterial catheters	1.39	0.42	10.94	4.03	1.76∼9.19	0.001

CRKP-BSI, carbapenem-resistant *Klebsiella pneumoniae* bloodstream infection; CSKP-BSI, carbapenem-susceptible *Klebsiella pneumoniae* bloodstream infection.

### 3.4 The CSKP-BSI group compared to the control group

The clinical characteristics affecting the development of CSKP-BSI in patients were identified using univariate analyses and compared with those of control patients (Table 5). The factors determined to be more closely related with CSKP-BSI included liver diseases (OR = 2.01; 95%CI, 1.16–3.49; *p* = 0.01), malignancies (OR = 2.48; 95%CI, 1.28–4.80; *p* = 0.006); surgical history (OR = 2.24; 95%CI, 1.27–3.94; *p* = 0.005), and invasive procedures, including central venous catheter insertion (OR = 2.27; 95%CI, 1.30–3.95; *p* = 0.004), within 1 month before a positive blood culture. Multivariate conditional logistic regression analysis indicated that liver diseases (OR = 1.82; 95%CI, 1.03–3.22; *p* = 0.04), and malignancies (OR = 2.38; 95%CI, 1.20–4.75; *p* = 0.01) were independent risk factors for CSKP-BSI (Table 4).

**TABLE 5:** Univariate analyses of the risk factors for CSKP-BSI compared to the control group (*N* = 105).

Variable	CSKP [n (%)]	Control [n (%)]	χ^2^/U	OR	95%CI	*p*
**Demographic characteristics**						
Sex, male	67 (63.8)	65 (61.9)	0.08	1.03	(0.84, 1.27)	0.78
**Underlying disorder**						
Respiratory diseases	67 (63.8)	66 (62.9)	0.02	1.04	(0.59, 1.83)	0.89
Liver diseases	66 (62.9)	48 (45.7)	6.22	2.01	(1.16, 3.49)	0.01
Urinary system diseases	47 (44.8)	49 (46.7)	0.08	0.93	(0.54, 1.59)	0.78
Circulatory diseases	69 (65.7)	78 (74.3)	1.84	0.66	(0.37, 1.20)	0.18
Central nervous diseases	35 (33.3)	37 (35.2)	0.09	0.92	(0.52, 1.63)	0.77
Digestive system diseases	14 (13.3)	22 (21.0)	2.15	0.58	(0.28, 1.21)	0.14
Diabetes mellitus	38 (36.2)	37 (35.2)	0.02	1.04	(0.59, 1.83)	0.89
Malignancies	34 (32.4)	17 (16.2)	7.48	2.48	(1.28, 4.80)	0.006
**Antibiotics exposure within 3 months before CRKP-BSI**						
3rd-generation cephalosporins	26 (24.8)	30 (28.6)	0.39	0.82	(0.45, 1.52)	0.53
β-lactam inhibitor	54 (51.4)	53 (50.5)	0.02	1.04	(0.61, 1.79)	0.89
Carbapenem	19 (18.1)	26 (24.8)	1.39	0.67	(0.35, 1.31)	0.24
Aminoglycosides	1 (1.0)	3 (2.9)	1.02	0.33	(0.03, 3.20)	0.31
Quinolones	16 (15.2)	17 (16.2)	0.04	0.93	(0.44, 1.96)	0.85
Tigecycline	14 (13.3)	14 (13.3)	0	1.00	(0.45, 2.22)	1
Glycopeptides	6 (5.7)	2 (1.9)	2.08	3.12	(0.62, 15.83)	0.15
Antifungal agents	12 (11.4)	13 (12.4)	0.05	0.91	(0.40, 2.11)	0.83
Glucocorticoid	22 (21.0)	23 (21.9)	0.03	0.95	(0.49, 1.83)	0.87
**Surgical history and invasive procedures within 1 month before CRKP-BSI**						
Surgical history	52 (49.5)	32 (30.5)	7.94	2.24	(1.27, 3.94)	0.005
Mechanical ventilation	40 (38.1)	36 (34.3)	0.33	1.18	(0.67, 2.07)	0.57
Central venous catheter insertion	68 (64.8)	47 (44.8)	8.48	2.27	(1.30, 3.95)	0.004
Arterial catheters	42 (40.0)	44 (41.9)	0.08	0.92	(0.53, 1.60)	0.78
Urinary catheter insertion	58 (55.2)	59 (56.2)	0.02	0.96	(0.56, 1.66)	0.89
Drainage tube insertion	21 (20.0)	16 (15.2)	0.82	1.39	(0.68, 2.84)	0.37
Gastric tube insertion	50 (47.6)	49 (46.7)	0.02	1.04	(0.60, 1.79)	0.89
**Related to hospitalization**						
Prior hospital stays median (IQR)	12 (5, 22)	12 (4, 25.5)	5707	—	—	0.66
ICU stay, median (IQR)	0 (0, 11)	0 (0, 15)	6009	—	—	0.22
LOS (days), median (IQR)	32 (18, 50.5)	34 (16, 70)	5857	—	—	0.43
Death	30 (28.6)	26 (24.8)	0.39	1.22	(0.66, 2.24)	0.53

For non-normally distributed quantitative data, they were expressed as median (IQR); Qualitative data were expressed as number and percentage (n (%)). β-lactam inhibitor referred to β-lactamase inhibitors (piperacillin tazobactam and cefoperazone sulbactam), and carbapenem referred to one of the carbapenems (imipenem, meropenem, doripenem). CSKP-BSI, carbapenem-susceptible *Klebsiella pneumoniae* bloodstream infection; ICU, intensive care unit; LOS, length of hospital stays; IQR, interquartile range; —, no result.

### 3.5 Clinical outcomes

**FIGURE 3:**
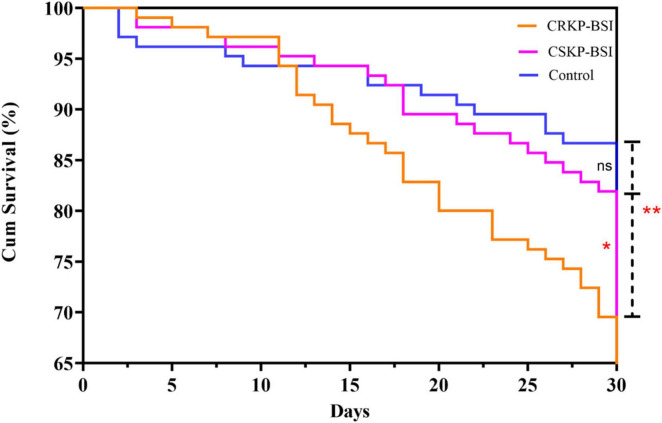
Kaplan–Meier curves showing 30-day mortality for the CRKP-BSI group, CSKP-BSI group and control group (*p* = 0.007). A significant difference was observed between the CRKP-BSI group and the CSKP-BSI group (*p* = 0.04) as well as between the CRKP-BSI group and the control group (*p* = 0.004). However, there was no significant difference between the CSKP-BSI group and the control group (*p* = 0.37).

Overall, the 30-day mortality of patients with CRKP-BSI was 30.5% (32/105), while that of CSKP-BSI patients was 18.1% (19/105), and that of the control group was 13.3% (14/105), The 30-day mortality was significantly higher in the CRKP-BSI group compared to both the CSKP-BSI group and the control group (*p* = 0.007). A significant difference was also observed in the length of hospital stays among the study groups (*p* = 0.01). The clinical outcomes of the three groups are listed in Table 6. The variables associated with 30-day mortality in the Cox hazard model were patient groups (*p* = 0.02), respiratory diseases (HR = 3.52; 95%CI, 1.37–9.06; *p* = 0.009); use of 3rd-generation cephalosporins (HR = 1.92; 95%CI, 1.10–3.33; *p* = 0.02); mechanical ventilation (HR = 2.14; 95%CI, 1.15–3.97; *p* = 0.02); central venous catheter insertion (HR = 2.85; 95%CI, 1.46–5.59; *p* = 0.002) and length of hospital stays (HR = 0.90; 95%CI, 0.88–0.92; *p* < 0.001). Kaplan-Meier survival analysis was performed, and the results are shown in Figure 3.

**TABLE 6:** Clinical outcomes comparison between CRKP-BSI, CSKP-BSI, and control groups.

Outcomes	CRKP-BSI [n (%)]	CSKP-BSI [n (%)]	Control [n (%)]	*p*
30-day mortality	32 (30.5)	19 (18.1)	14 (13.3)	0.007
In-hospital mortality	58 (55.2)	30 (28.6)	26 (24.8)	<0.001
LOS (days), median (IQR)	41 (20, 66)	32 (18, 50.5)	34 (16, 70)	0.01

CRKP-BSI, carbapenem-resistant *Klebsiella pneumoniae* bloodstream infection; CSKP-BSI, carbapenem-susceptible *Klebsiella pneumoniae* bloodstream infection; LOS, length of hospital stays; IQR, interquartile range.

Fifty-eight patients (55.2%) in the CRKP-BSI group, 30 patients (28.6%) in the CSKP-BSI group, and 26 patients (24.8%) in the control group died during hospitalization, in-hospital mortality was higher in the CRKP-BSI group compared to both the CSKP-BSI group and the control group (*p* < 0.001). Univariate analyses comparing the clinical characteristics of patients with CRKP-BSI who survived or died are presented in Table 7. Univariate analysis indicated that arterial catheter use (OR = 3.76; 95%CI, 1.67–8.49; *p* = 0.001), and ICU stays (*p* = 0.006) were associated with in-hospital mortality. Multivariate conditional logistic regression analysis showed that arterial catheter placement within 1 month before a positive blood culture (OR = 3.76; 95%CI, 1.67–8.49; *p* = 0.001) was an independent risk factor for in-hospital mortality (Table 4).

**TABLE 7:** Univariable analyses of in-hospital mortality in the CRKP-BSI group.

Variable	CRKP-BSI death (*N* = 58) [n (%)]	CRKP-BSI survivors (*N* = 47) [n (%)]	χ^2^/U	OR	95%CI	*p*
**Demographic characteristics**						
Sex, male	45 (77.6)	31 (66.0)	1.76	1.79	(0.75, 4.24)	0.19
Age (years), median (IQR)	72 (61, 83)	68 (54, 76)	1159	–	–	0.19
Age > 65	36 (62.1)	29 (61.7)	0.001	1.02	(0.46, 2.24)	0.97
**Underlying disorder**						
Respiratory diseases	50 (86.2)	35 (74.5)	2.32	2.14	(0.79, 5.79)	0.13
Liver diseases	38 (65.5)	25 (53.2)	1.64	1.67	(0.76, 3.68)	0.20
Urinary system diseases	32 (55.2)	23 (48.9)	0.41	1.28	(0.59, 2.78)	0.53
Circulatory diseases	40 (69.0)	30 (63.8)	0.31	1.26	(0.56, 2.84)	0.58
Central nervous diseases	24 (41.4)	14 (29.8)	1.51	1.66	(0.74, 3.76)	0.22
Digestive system diseases	17 (29.3)	14 (29.8)	0.003	0.98	(0.42, 2.27)	0.96
Diabetes mellitus	17 (29.3)	13 (27.7)	0.04	1.08	(0.46, 2.55)	0.85
Malignancies	13 (22.4)	17 (36.2)	2.41	0.51	(0.22, 1.20)	0.12
**Antibiotics exposure within 3 months before CRKP-BSI**						
3rd-generation cephalosporins	25 (43.1)	19 (40.4)	0.08	1.12	(0.51, 2.44)	0.78
β-lactam inhibitor	41 (70.7)	32 (68.1)	0.08	1.13	(0.49, 2.60)	0.77
Carbapenem	43 (74.1)	30 (63.8)	1.30	1.62	(0.70, 3.75)	0.25
Quinolones	20 (34.5)	14 (29.8)	0.26	1.24	(0.54, 2.84)	0.61
Tigecycline	27 (46.6)	13 (27.7)	3.93	2.28	(1.00, 5.18)	0.05
Macrolides	1 (1.7)	0 (0)	0.82	–	–	0.37
Glycopeptides	7 (12.1)	3 (6.4)	0.97	2.01	(0.49, 8.26)	0.32
Antifungal agents	14 (24.1)	8 (17.0)	0.79	1.55	(0.59, 4.09)	0.37
Glucocorticoid	32 (55.2)	23 (48.9)	0.41	1.28	(0.59, 2.78)	0.53
**Surgical history and invasive procedures within 1 month before CRKP-BSI**						
Surgical history	28 (48.3)	27 (57.4)	0.88	0.69	(0.32, 1.50)	3.45
Mechanical ventilation	40 (69.0)	24 (51.1)	3.50	2.13	(0.96, 4.73)	0.06
Central venous catheter insertion	47 (81.0)	33 (70.2)	1.68	1.81	(0.73, 4.49)	0.20
Arterial catheters	37 (63.8)	15 (31.9)	10.55	3.76	(1.67, 8.49)	0.001
Urinary catheter insertion	47 (81.0)	33 (70.2)	1.68	1.81	(0.73, 4.49)	0.20
Drainage tube insertion	19 (32.8)	13 (27.7)	0.32	1.27	(0.55, 2.96)	0.57
Gastric tube insertion	41 (70.7)	27 (57.4)	1.99	1.79	(0.80, 4.01)	0.16
**Related to hospitalization**						
Prior hospital stays median (IQR)	25 (14, 48)	21 (11, 43)	1234	–	–	0.40
ICU stays, median (IQR)	16 (5, 35)	0 (0, 31.75)	944	–	–	0.006
LOS (days), median (IQR)	35 (19, 58)	49.5 (28, 82)	1596	–	–	0.13
**Antimicrobial therapy**						
Untreated	2 (3.4)	1 (2.1)	0.16	1.64	(0.14, 18.7)	0.69
Monotherapy with tigecycline	6 (10.3)	5 (10.6)	0.002	0.97	(0.28, 3.40)	0.96
Monotherapy with colistin	0 (0)	1 (2.1)	1.25	–	–	0.26
Monotherapy without tigecycline or colistin	21 (36.2)	17 (36.2)	0.00	1.00	(0.45, 2.23)	1.00
Combine therapy without tigecycline or colistin	5 (8.6)	2 (4.3)	0.80	2.12	(0.39, 11.47)	0.37
Combine therapy with tigecycline	10 (17.2)	10 (21.3)	0.27	0.77	(0.29, 2.05)	0.60
Combine therapy with colistin	3 (5.2)	2 (4.3)	0.05	1.23	(0.20, 7.67)	0.83
Combine therapy with tigecycline and colistin	4 (6.9)	4 (8.5)	0.10	0.80	(0.19, 3.37)	0.76
Ceftazidime/avibactam	7 (12.1)	5 (10.6)	0.05	1.15	(0.34, 3.90)	0.82

For non-normally distributed quantitative data, they were expressed as median (IQR); Qualitative data were expressed as number and percentage [n (%)]. β-lactam inhibitor referred to β-lactamase inhibitors (piperacillin tazobactam and cefoperazone sulbactam), and carbapenem referred to one of the carbapenems (imipenem, meropenem, doripenem). SD, standard deviation; CRKP-BSI, carbapenem-resistant *Klebsiella pneumoniae* bloodstream infection; ICU, intensive care unit; LOS, length of hospital stays; IQR, interquartile range; –, no result.

## 4 Discussion

This study provides important insights into the characteristics and outcomes of CRKP in BSIs. For studies on risk factors for CRKP infection, the selection of an appropriate control group is critical to generate correct conclusions. Several studies selected the CSKP group as the control group, which can introduce statistical selection bias. CSKP represents only a small proportion of nosocomial infections and may not be representative of all infections. In addition, certain antibiotics may inhibit or eliminate certain strains of CSKP, while not affecting CRKP, which is another important bias. Consequently, the frequency of antibiotic use in CSKP strains may decrease, indirectly amplifying the risk factors for CRKP (Ng et al., 2014). Alternatively, a few studies have only included patients who were not infected with CRKP as controls but did not distinguish between susceptible and resistant groups (Cronin et al., 2017; Giacobbe et al., 2017; Micozzi et al., 2017). However, the risk factors for KP infection cannot be analyzed in such a scenario. Therefore, we performed a case-case-control study that included matched control from inpatients to identify the risk factors for CRKP/CSKP infection and the 30-day mortality of CRKP, thereby guiding the prevention and appropriate hospital treatment strategies.

In our study, we determined that CRKP-BSI was primarily concentrated in the ICU, followed by the hematology and emergency departments in the general medical department. Nosocomial infections are readily acquired in the ICU due to airborne and contact transmission of CRKP in this confined environment (Jiao et al., 2015). The transmission of CRKP is facilitated in the ICU by frequent use of medical equipment and invasive procedures. Patients in the ICU have serious underlying diseases and may be treated with broad-spectrum antibiotics, which also contribute to the induction of CRKP-BSI. Notably, hematology patients present unique vulnerabilities that increase the risk of CRKP-BSI, including long-term hospitalization, use of high-grade antibiotics, and an impaired immune response (Chen J. et al., 2022; de Souza et al., 2024). Patients with various infectious diseases are often treated in the emergency department, where crowdedness with patients, heavy workload of medical staff, and inadequate implementation of infection control measures are common problems that may lead to cross-transmission of CRKP in the emergency department.

The findings of this study showed that antibiotic resistance was more pronounced in the CRKP-BSI group than that in the CSKP-BSI group. However, these strains remained relatively susceptible to colistin and tigecycline. Considering the above results and individual clinical conditions, combination therapy is recommended as the optimal treatment approach for CRKP-BSI.

The statistically significant factors identified in multivariate analyses of both the CRKP-BSI and CSKP-BSI groups were risk factors for *K. pneumoniae*-BSI. The factors that were statistically significant in the multivariate analysis of CRKP-BSI but not of CSKP-BSI were risk factors for CRKP-BSI. In contrast, factors that were statistically significant in the multivariate analysis of CSKP-BSI but not of CRKP-BSI were risk factors for CSKP-BSI (Kaye et al., 2005). We identified that exposure to antibiotics (carbapenems and tigecycline) and glucocorticoids were risk factors for CRKP-BSI. One risk factor, liver disease, was a risk factor for *K. pneumoniae*-BSI. Patients with liver diseases have altered enteric flora and impaired immune systems, which increase the risk of *K. pneumoniae*-BSI (Hsu et al., 2021; Lameirão Gomes et al., 2019). In addition, patients with liver diseases require invasive procedures for diagnosis and treatment, which can exacerbate the occurrence of nosocomial infections.

Consistent with the results of previous studies (Cao et al., 2022; Xiao et al., 2020), our study showed that prior carbapenem use was an independent risk factor for CRKP-BSI. Carbapenemase acquisition is the primary cause of carbapenem resistance and occurs in across diverse departments (Zhang et al., 2021). Han et al. showed that the most prevalent carbapenemase gene in CRKP in China is blaKPC-2, followed by blaNDM (Han et al., 2020), and carbapenem exposure may induce the emergence of resistance-conferring genes. In contrast to other studies, our study determined that tigecycline exposure was also a risk factor for CRKP-BSI, indicating that CRKP-BSI can be induced by carbapenem as well as by other drugs. A previous study suggested that overexpression of the AcrAB efflux pump, induced by activated MarA, plays an important role in the emergence of CRKP isolates (Park et al., 2020). The use of antibiotics within 3 months before a positive blood culture indicated that the patient may have had repeated infections and antibiotic exposure, thereby increasing the risk of antibiotic resistance development. Thus, there is a need to strengthen the management of antibiotics in inpatients. Treatment with high doses for appropriate durations is a better way to limit the risk of infection. Consistent with previous studies (Huang et al., 2023), we showed that glucocorticoids use is a risk factor for CRKP-BSI, possibly because patients with CRKP-BSI have serious underlying diseases and are treated with glucocorticoids. Second, the use of glucocorticoids destroys the intestinal microenvironment, kills susceptible strains, and promotes the overgrowth of drug-resistant strains, thereby promoting a shift from opportunistic pathogens to pathogenic bacteria. Therefore, more attention should be paid to the initial appropriate antibiotic therapy for patients with CRKP-BSI. In addition, strengthening antimicrobial stewardship programs and regular resistance surveillance is essential to avoid unnecessary antibiotic exposure.

When assessing the potential impact of CRKP-BSI on 30-day mortality, we determined that patient groups, respiratory diseases, use of 3rd-generation cephalosporins, mechanical ventilation, and central venous catheter insertion were independent risk factors for 30-day mortality. However, a shorter length of hospital stays was a protective factor against 30-day mortality. Various studies have reported that mortality in patients with CRKP-BSI ranged from 24 to 65%, and some studies have shown that CRKP infection is an independent risk factor for mortality (Cao et al., 2022; Chen J. et al., 2022; Li et al., 2020; Liu et al., 2019; Schwaber et al., 2008). Similarly, in our study, patients with CRKP-BSI exhibited higher mortality and longer hospital stays.

Our study showed that patients with nosocomial CRKP-BSI were often diagnosed with respiratory diseases, which has been reported in previous studies (Strich et al., 2020). Moreover, several studies have also shown that chronic obstructive pulmonary disease (COPD) is a risk factor for CRKP infection (Liu et al., 2012; Nouvenne et al., 2014; Salsano et al., 2016). Patients with respiratory diseases are more likely to be infected with or colonized by CRKP in the airway, and patients with respiratory diseases requiring mechanical ventilation or invasive procedures are susceptible to CRKP-BSI. Under the selective pressure of 3rd-generation cephalosporin use, susceptible strains are eliminated, thereby facilitating the survival of resistant strains. Mechanical ventilation and central venous catheter insertion were independent risk factors for 30-day mortality in patients with CRKP-BSI. The most likely reason for this is that invasive procedures can damage the mucosa and increase the incidence of CRKP-BSI because the majority of bacteria can pass through the mucosal barrier to the bloodstream (Li et al., 2020). A shorter LOS is a protective factor against 30-day mortality. Patients with a reduced LOS have a lower risk of CRKP infection due to decreased contact time with the hospital environment, medical staff, and other patients, which is consistent with the findings of previous studies (Correa et al., 2013; Gallagher et al., 2014). The longer the hospital stays, the greater the risk of infection from colonizing bacteria. Therefore, judicious use of 3rd-generation cephalosporins in antibiotic stewardship programs, reduction of invasive procedures, and prevention of CRKP-BSI are effective strategies to reduce 30-day mortality.

Not surprisingly, arterial catheterization was a risk factor for in-hospital mortality in patients with nosocomial CRKP-BSI, which is consistent with previous studies emphasizing the importance of aseptic operations in patient care, and that invasive procedures can damage the mucosa and increase the chance of CRKP entering the bloodstream (Wang et al., 2018). Maintaining aseptic technique is an important strategy for CRKP-BSI prevention.

Recently, an increasing number of studies have focused on active screening for CRKP to enable early detection, isolation, and intervention in high-risk departments and patients. The WHO and the US CDC recommend screening for CRE based on local epidemiological conditions (United States Department of Health and Human Services, 2015; World Health Organization, 2017). In our hospital, nosocomial CRKP-BSIs primarily occur in the ICU and in patients with specific risk factors, suggesting that higher-risk departments and patients should be actively screened for CRKP.

Therapeutic options for CRKP-BSI in our hospital include these based on monotherapy or, in combination, tigecycline, colistin or ceftazidime/avibactam. Outcomes of patients were not altered by different types of antimicrobial therapy in univariate analysis, which was consistent with previous studies (Xiao et al., 2018). This is mainly attributes to the optimal treatment option for CRKP infections is still not well established. The causes of death in patients with CRKP-BSI are complex. Clinicians should not only consider anti-infective treatment, but also consider the patient’s own conditions, including the patient’s underlying diseases and organ functions. The prompt identification of risk factors for mortality in patients infected with CRKP-BSI is essential for early prescription of adequate empirical antibiotic therapy, which was a key factor for mortality reduction.

The present study has several limitations. First, this was a single-center study with a small sample size. Therefore, it is imperative to conduct prospective, multicenter, large-sample clinical trials to validate our findings. Second, this was a retrospective cohort, and potential information and selection biases were unavoidable. Finally, the clinical microbiology laboratory of our hospital does not perform routine detection of carbapenemase production for CRKP. We cannot exclude the possibility that some carbapenemase-producing isolates were susceptible to carbapenems and may have been classified as CSKP in this study. We also could not assess whether any outbreaks occurred during the study period and whether different drug resistance mechanisms resulted in different clinical outcomes. Future studies should include molecular epidemiological investigations.

## 5 Conclusion

This study aimed to elucidate the predictors of CRKP-BSI and help clinicians better identify CRKP-BSI at an early stage. In our hospital, CRKP-BSI exhibited a focal distribution across specific departments, such as the ICU, hematology department, and emergency department, highlighting the need to implement interventions such as hand hygiene protocols and environmental disinfection measures to limit CRKP transmission. The identification of risk factors for CRKP-BSI infection and mortality are critical for preventing and controlling CRKP. Our results showed that liver disease is an independent risk factor for *K. pneumoniae*-BSI, and that exposure to antibiotics (carbapenems and tigecycline) and glucocorticoids are risk factors for CRKP-BSI. central venous catheter insertion and mechanical ventilation use were independently associated with 30-day mortality. Therefore, patients with CRKP-BSI, respiratory diseases, use of 3rd-generation cephalosporins, mechanical ventilation, central venous catheter insertion, and arterial catheter use are more likely to have a poor prognosis. Close attention should be paid to patients who have used carbapenem and tigecycline over an extended period. Moreover, outcomes of patients were not altered by different types of antimicrobial therapy, the prompt identification of risk factors for mortality was a key factor for mortality reduction. The rational use of antibiotics, reduction in the length of hospital stays and reducing the frequency and duration of invasive procedures are effective measures to reduce CRKP-BSI and 30-day or in-hospital mortality (Xiao et al., 2018; Yuan et al., 2020). These findings provide valuable insights for the development of anti-infective therapy, infection prevention and the control strategies for CRKP-BSIs.

## Data Availability

The original contributions presented in the study are included in the article/supplementary material, further inquiries can be directed to the corresponding authors.
